# Patterns and Associated Factors of Breakthrough Pain in Lung Cancer: Insights From a Cross‐Sectional Study With Prospective Enrollment

**DOI:** 10.1155/prm/2783248

**Published:** 2026-08-19

**Authors:** Xiao-Meng Dou, Bao-Wen Huang, Xiao-Xiao Huang, Jin-Bo Li, Jiu-Di Zhong, Hui-Ying Qin

**Affiliations:** ^1^ Department of Thoracic Surgery, Sun Yat-Sen University Cancer Center, Guangzhou, China, sysucc.org.cn; ^2^ State Key Laboratory of Oncology in South China, Collaborative Innovation Center for Cancer Medicine, Sun Yat-Sen University Cancer Center, Guangzhou, China, sysucc.org.cn; ^3^ Department of Nursing, Sun Yat-Sen University Cancer Center, Guangzhou, China, sysucc.org.cn

**Keywords:** anxiety, breakthrough pain, cough, lung cancer

## Abstract

**Background:**

Breakthrough cancer pain (BTcP) is a challenging symptom in lung cancer patients, often associated with poor physical and psychological outcomes. Understanding its clinical features and associated factors is essential for improving pain management and patient care.

**Methods:**

This cross‐sectional study prospectively enrolled lung cancer patients with BTcP treated at our center between October 2024 and January 2025. Data were collected through questionnaires and medical records, including demographic, clinical, psychological, and symptom‐related variables. Logistic regression analysis was conducted to identify factors associated with BTcP severity.

**Results:**

A total of 255 eligible patients were enrolled. BTcP was most commonly located in the chest (148/255, 58.0%) and frequently triggered by coughing (124/255, 48.6%). Most episodes were of short duration and moderate to severe intensity. Multivariate analysis identified treatment modality, cough VAS score, and anxiety level as independent associated factors of BTcP severity (*p* < 0.05). In the postoperative subgroup (*n* = 159), surgical extent, number of drain tube, cough VAS score, and anxiety level were significant factors for BTcP severity.

**Conclusions:**

BTcP in lung cancer patients is characterized by high frequency and moderate‐to‐severe intensity, with anxiety and cough being key contributing factors. Comprehensive pain management should integrate psychological support and symptom control to improve patient outcomes.

## 1. Introduction

Lung cancer remains a leading malignancy worldwide in both incidence and mortality [1, 2]. The early symptoms of lung cancer are often insidious, leading to most patients being diagnosed at an advanced stage [3]. The majority of patients with advanced lung cancer experience disease‐related symptoms, among which pain is one of the most common [4]. Most of patients with advanced lung cancer experience pain due to distant metastases to organs such as bone, local tumor invasion into structures such as the chest wall, or tumor mass–related nerve compression, with over half reporting moderate to severe pain [5].

Breakthrough cancer pain (BTcP) is one of the most challenging forms of pain to manage in advanced lung cancer [6, 7]. It is typically characterized by transient exacerbations of pain that occur despite otherwise controlled baseline pain, triggered either spontaneously or by predictable or unpredictable factors [6]. The World Health Organization (WHO), in its Guidelines for the Management of Cancer Pain, explicitly identifies BTcP as a key focus in cancer pain management [8]. A meta‐analysis reported an overall pooled BTcP prevalence of 59.2%, ranging from 39.9% in outpatient clinics to 80.5% in hospice care [9]. Importantly, lung cancer represents a clinically relevant subgroup of BTcP. A secondary analysis of the IOPS‐MS study focusing on 1,087 lung cancer patients with BTcP showed that these patients had higher background pain intensity, more severe BTcP episodes, greater interference with daily activities, and predictable triggers such as movement and cough [10]. These findings underscore the clinical importance of disease‐specific assessment and management of BTcP in lung cancer patients.

BTcP causes severe distress in lung cancer patients and can lead to debilitation, anxiety, and depression, markedly reducing daily function and quality of life [11]. In clinical practice, the management of BTcP has become an integral part of oncology nursing, with nurses playing a key role in pain assessment, intervention, and patient education [12]. However, research on the characteristics of BTcP in lung cancer patients remains in its early stages, with a lack of systematic clinical evidence. This study analyzed clinical data from lung cancer patients with BTcP at a high‐volume cancer center to identify key characteristics and influencing factors, aiming to support improved identification and targeted clinical interventions.

## 2. Materials and Methods

### 2.1. Patient Selection

This cross‐sectional study prospectively enrolled lung cancer patients treated between October 2024 and January 2025 in the department of thoracic surgery, medical oncology, and radiation oncology at our cancer center. The inclusion criteria were as follows: (1) age ≥ 18 years; (2) clinically or pathologically confirmed diagnosis of primary lung cancer; (3) meeting the diagnostic criteria for BTcP; and (4) voluntary participation with informed consent signed by the patient and their family. Exclusion criteria included the following: (1) the presence of other serious physical illnesses, such as serious cardiovascular disease, severe hepatic or renal dysfunction, severe respiratory disease, or other major medical conditions that could interfere with study participation; and (2) impaired consciousness or a documented history of intellectual, psychiatric, or cognitive disorders.

The screening process was conducted by trained research nurses in collaboration with the clinical care team. First, patients with a confirmed diagnosis of primary lung cancer were identified through a daily review of admission records, diagnosis lists, treatment records, and nursing pain assessment records. Patients with documented cancer‐related pain, baseline pain scores, or analgesic use were further flagged as potentially eligible for BTcP assessment. Second, the research nurses reviewed the medical records to collect information on diagnosis, tumor stage, current treatment status, baseline pain intensity, and ongoing analgesic therapy. Third, a bedside interview was conducted to confirm the presence of persistent background pain during the previous week, the adequacy of background pain control, and the occurrence of transient pain exacerbations. During the interview, patients were specifically asked about the intensity, duration, frequency, triggers, and relieving factors of pain flares.

BTcP was diagnosed with reference to the criteria proposed by Davies et al. [13] and based on the following conditions: (1) the presence of persistent background pain during the previous week; (2) adequate control of background pain during the previous week, defined as a numeric rating scale (NRS) score of ≤ 3; and (3) the presence of transient pain exacerbations with an NRS score of ≥ 4. Background pain control was assessed using the recorded pain score together with information on ongoing analgesic treatment from the medical records and patient interview. BTcP was distinguished from uncontrolled background pain by identifying transient pain flares superimposed on otherwise controlled background pain. No formal protocol for optimization of background analgesia before enrollment was applied.

### 2.2. Ethical Approval

This study was approved by the Ethics Committee of the Sun Yat‐Sen University Cancer Center (Ethics Approval No. B2025‐072‐01, Document S1) and received support and consent from the relevant departments. Written informed consent was obtained only after ensuring full patient understanding. Participants were guaranteed the right to informed participation and voluntary withdrawal at any stage of the study. Questionnaires were completed anonymously, and all procedures adhered strictly to confidentiality principles. Researchers maintained the privacy of all patient information, and data were used solely for the analysis of clinical characteristics and influencing factors of BTcP in lung cancer patients, with no other purposes. This study was conducted in accordance with the Declaration of Helsinki [14].

### 2.3. Data Collection

Data collection in this study was primarily conducted through questionnaires and medical record review. The research tools included a general information questionnaire, clinical data form, the original English version of the Breakthrough Pain Assessment Tool (BAT) [15], the visual analog scale (VAS) [16], and the Hospital Anxiety and Depression Scale (HADS) [17]. The general information questionnaire, developed by the researchers, gathered demographic and disease‐related data, including gender, age, body mass index (BMI), marital status, ethnicity, religion, educational level, occupation, household income, smoking and drinking history, medical history, use of analgesics, and treatment modalities. The clinical data form recorded medical details such as diagnosis, tumor stage, pathological type, presence of metastasis, treatment methods, baseline pain score, analgesic usage, cough intensity score, and white blood cell count. BTcP was assessed using the BAT, which comprises 16 items [15]. Ten items specifically address characteristics of BTcP in the past week, including pain location, frequency, triggers, relieving factors, duration, peak pain intensity, average pain intensity, level of distress during pain episodes, and interference with daily life [15]. Patients rated pain intensity, distress, and disruption to daily life on a numeric scale from 0 to 10, with higher scores indicating greater severity [15]. The remaining six items relate to pain management, covering analgesic type, effectiveness, onset of action, presence of side effects, and the degree of distress caused by those side effects [15]. BTcP severity was further categorized as moderate (NRS 4–6) and severe (NRS 7–10). Cough severity was evaluated using the VAS, which is simple to administer and highly practical. Patients marked a point on a 0–10‐cm or 0–100‐mm line that best represented their perceived cough severity. Anxiety and depression were assessed using the HADS.

### 2.4. Statistical Analysis

Data were entered independently by two researchers using a double‐entry method, with real‐time verification and correction to ensure accuracy. Statistical analysis was performed using SPSS Version 25.0 and R statistics Version 4.3.2. A *p* value < 0.05 was considered statistically significant. Continuous variables were presented as medians with interquartile ranges (IQRs), while categorical variables were expressed as frequencies and percentages. Univariate and multivariate logistic regression were used to identify independent factors associated with the severity of BTcP. Variance inflation factor (VIF) analysis was conducted to assess multicollinearity among independent variables.

## 3. Results

### 3.1. Clinicopathologic Characteristics of Included Patients

A total of 255 lung cancer patients with BTcP were enrolled in this study, including 148 males (148/255, 58.0%) and 107 females (42.0%). The median age was 58 years (IQR: 48–66). Most patients had adenocarcinoma (63.9%), and nearly half were diagnosed at Stage I (45.9%). Surgical resection was the most common treatment (62.4%). All patients had background pain, with 149 reporting a baseline pain score of 2 (58.4%). Regarding symptom‐related characteristics, cough was predominantly mild to moderate, affecting 120 (47.1%) and 83 (32.5%) patients, respectively. Anxiety was absent or mild in most patients, accounting for 117 (45.9%) and 87 (34.1%) patients, respectively. The detailed data are presented in Table 1.

**TABLE 1:** The general clinicopathological characteristics of included patients (*n* = 255).

Characteristics	*n* (%)
*Age, years*	
Median (IQR)	58 (48–66)

*Gender*	
Male	148 (58.0)
Female	107 (42.0)

*Religion*	
Without	248 (97.3)
With	7 (2.7)

*Ethnicity*	
Han	247 (96.9)
Others	8 (3.1)

*Marital status*	
Married	233 (91.4)
Others	22 (8.6)

*Drinking history*	
Without	242 (94.9)
With	13 (5.1)

*Smoking*	
Never smoker	151 (59.2)
Former smoker	89 (34.9)
Current smoker	15 (5.9)

*BMI*	
Median (IQR)	23.2 (21.2–25.5)

*Educational level*	
Primary school or below	52 (20.4)
Junior high school	83 (32.5)
Vocational school	24 (9.4)
Senior high school	33 (12.9)
College diploma	23 (9.0)
Bachelor degree	36 (14.1)
Postgraduate degree	4 (1.6)

*Residence*	
Urban	156 (61.2)
Rural	99 (38.8)

*Occupation*	
Retired/unemployed	76 (29.8)
Employed	179 (70.2)

*Income, yuan*	
≤ 2000	34 (13.3)
2000–4000	70 (27.5)
4000–6000	52 (20.4)
≥ 6000	99 (38.8)

*Hypertension*	
Without	197 (77.3)
With	58 (22.7)

*Diabetes mellitus*	
Without	222 (87.1)
With	33 (12.9)

*Tumor site*	
Right	151 (59.2)
Left	81 (31.8)
Others	23 (9.0)

*Treatment modality*	
Surgery	159 (62.4)
Chemotherapy	78 (30.6)
Other	18 (7.1)

*Pathological type*	
Adenocarcinoma	163 (63.9)
Squamous cell carcinoma	40 (15.7)
Others	52 (20.4)

*TNM stage*	
I	117 (45.9)
II	10 (3.9)
III	28 (11.0)
IV	77 (30.2)
Undetermined	23 (9.0)

*Baseline pain score*	
1	17 (6.7)
2	149 (58.4)
3	89 (34.9)

*Cough severity*	
No (score 0)	17 (6.7)
Mild (score 1–3)	120 (47.1)
Moderate (score 4–6)	83 (32.5)
Severe (score 7–9)	35 (13.7)
Extreme (score 10)	0 (0.0)

*Anxiety assessment*	
No	117 (45.9)
Mild	87 (34.1)
Moderate	32 (12.5)
Severe	19 (7.5)

*Depression assessment*	
No	108 (42.4)
Mild	77 (30.2)
Moderate	46 (18.0)
Severe	24 (9.4)

*Note:* IQR, interquartile range.

### 3.2. Characterization of BTcP in Lung Cancer Patients

Among lung cancer patients with BTcP, the chest was the most frequently reported pain site (58.0%), followed by the lower limbs (16.5%) and back (11.8%). The frequency of BTcP episodes was most commonly 1–2 times per day (44.7%), followed by more than 4 times per day (25.1%) and 3–4 times per day (20.8%). Cough was the most frequently reported trigger (48.6%), followed by repositioning (20.8%) and physical contact (11.4%). The most common time of day for BTcP episodes was between 10:00 a.m. and 6:00 p.m. (51.8%), followed by 02:00 a.m–10:00 a.m (34.1%) and 6:00 p.m–02:00 a.m (14.1%). The majority of pain episodes lasted less than 5 min (42.4%). The most intense breakthrough pain was rated as severe in 147 patients (57.6%) and moderate in 108 patients (42.4%). BTcP was most commonly described as sharp or stabbing (45.5%), followed by pressure‐like and diffuse pain (11.4%). Psychological distress caused by BTcP had a median score of 5 (IQR: 3–8), while interference with daily life had a median score of 6 (IQR: 4–8). A total of 160 cases (62.7%) used analgesic medications for pain relief. Among those receiving medication, weak opioids were the most commonly used (69/160, 27.1%), followed by nonsteroidal anti‐inflammatory drugs (54/160, 21.2%) and strong opioids (37/160, 14.5%). Additionally, 115 patients (115/255, 45.1%) employed nonpharmacological measures to manage pain, while 140 patients (140/255, 54.9%) did not. Regarding the onset of analgesic effects, 52.5% (84/160) reported pain relief after 30 min, 20.6% within 20–30 min, and 16.3% within 10–20 min. Only 2 patients reported no effect. The perceived effectiveness of analgesics had a median score of 7 (IQR: 5–8). In terms of side effects, 43.8% reported none. Constipation was the most common adverse effect (56/160, 35.0%), followed by dizziness (15.0%). The median score for distress caused by side effects was 3 (IQR: 0–5). Details are shown in Table 2.

**TABLE 2:** The features of breakthrough pain.

Item	*n* (%)
*Pain location*	
Head	11 (4.3)
Neck	11 (4.3)
Chest	148 (58.0)
Back	30 (11.8)
Abdomen	13 (5.1)
Lower limb	42 (16.5)

*Frequency per day*	
Less than 1 time	24 (9.4)
1–2 times	114 (44.7)
3–4 times	53 (20.8)
More than 4 times	64 (25.1)

*Triggers*	
None	11 (4.3)
Walking	26 (10.2)
Coughing	124 (48.6)
Touching	29 (11.4)
Turning over	53 (20.8)
Others	12 (4.7)

*Occurrence time period*	
10:00 a.m–6:00 p.m.	132 (51.8)
6:00 p.m–2:00 a.m.	36 (14.1)
2:00 a.m–10:00 a.m.	87 (34.1)

*General pain severity*	
Moderate (score 4–6)	145 (56.9)
Severe (score 7–10)	110 (43.1)

*Maximum pain severity*	
Moderate (score 4–6)	108 (42.4)
Severe (score 7–10)	147 (57.6)

*Pain duration, min*	
Less than 5	108 (42.4)
5–15	48 (18.8)
15–30	24 (9.4)
30–60	34 (13.3)
More than 60	41 (16.1)

*Pain quality*	
Dull pain	22 (8.6)
Pressure pain	29 (11.4)
Stabbing pain	116 (45.5)
Colicky pain	16 (6.3)
Diffuse pain	29 (11.4)
Others	43 (16.9)

*Psychological distress level*	
Median (IQR)	5 (3–8)

*Impact on daily life severity*	
Median (IQR)	6 (4–8)

*Analgesic use*	
No	95 (37.3)
yes	160 (62.7)

*Analgesic drug type (n = 160)*	
Nonsteroidal anti‐inflammatory drugs	54 (21.2)
Weak opioids	69 (27.1)
Strong opioids	37 (14.5)

*Use of nonanalgesic measures*	
No	140 (54.9)
Yes	115 (45.1)

*Analgesic onset time,* min *(n = 160)*	
No analgesic effect	2 (1.3)
0–10	15 (9.4)
10–20	26 (16.3)
20–30	33 (20.6)
More than 30	84 (52.5)

*Adverse effects after analgesic (n = 160)*	
No	70 (43.8)
Constipation	56 (35.0)
Nausea	2 (1.3)
Vomiting	2 (1.3)
Dizziness	24 (15.0)
Pruritus	1 (0.6)
Urinary retention	5 (3.1)

*Analgesic efficacy (n = 160)*	
Median (IQR)	7 (5–8)

*Distress level of analgesic side effects*	
Median (IQR)	3 (0–5)

*Note:* IQR, interquartile range.

### 3.3. Analysis of Factors Associated With BTcP Severity in the Entire Cohort

The results indicated that tumor stage, treatment modality, cough VAS score, anxiety score, and depression score were potentially associated with the severity of BTcP (*p* < 0.05), whereas age, sex, BMI, and marital status showed no significant associations (*p* > 0.05) (Table S1). All the statistically significant variables had tolerance values greater than 0.1 and VIF values less than 5, indicating no serious multicollinearity (Table S2). After adjustment for potential confounders, treatment modality, cough VAS score, and anxiety score remained independently associated with BTcP severity (Table 3). Specifically, patients who received chemoradiotherapy had a significantly higher risk of experiencing severe BTcP compared to those who underwent surgery (OR = 3.493, 95% CI: 1.413–5.573, *p* < 0.001). Higher cough VAS scores were associated with increased pain severity (OR = 1.142, 95% CI: 1.001–1.305, *p* = 0.048). Additionally, patients with mild anxiety (OR = 2.273, 95% CI: 1.139–4.534, *p* = 0.020) and severe anxiety (OR = 20.706, 95% CI: 3.203–133.866, *p* < 0.001) had significantly higher odds of experiencing severe BTcP compared to those without anxiety.

**TABLE 3:** Multivariate logistic regression analysis of breakthrough pain severity in the entire cohort.

Characteristic	OR	95% CI	*p*
TNM stage			0.747
I	1		
II	1.401	0.315–6.237	
III	2.343	0.689–7.964	
IV	2.213	0.547–8.949	
Undetermined	1.215	0.438–3.368	
Treatment modality			**< 0.001**
Surgery	1		
Chemotherapy/radiotherapy	3.493	1.413–5.573	
Cough VAS score			**0.048**
Continual	1.142	1.001–1.305	
Anxiety assessment			**0.005**
No	1		
Mild	2.273	1.139–4.534	
Moderate	2.333	0.889–6.122	
Severe	20.706	3.203–133.866	
Depression assessment			0.894
No	1		
Mild	0.783	0.375–1.636	
Moderate	0.976	0.401–2.378	
Severe	0.711	0.149–3.391	

*Note:* Bold value means p < 0.05. Abbreviations: CI, confidence interval; OR, odds ratio; TNM, tumor–node–metastasis; VAS, visual analog scale.

### 3.4. Analysis of Factors Influencing BTcP in Postoperative Lung Cancer Patients

A total of 159 postoperative lung cancer patients with BTcP were included in this study. The extent of surgical resection, number of chest drains, cough VAS score, and anxiety score were significantly associated with the severity of BTcP in the initial analysis (*p* < 0.05) (Table S3). There was no severe multicollinearity among these significant variables (Table S4). After adjustment, the extent of surgical resection, number of chest drains, cough VAS score, and anxiety score remained independently associated with BTcP severity (Table 4). Specifically, patients who underwent lobectomy had a significantly higher risk of severe BTcP compared to those who underwent sublobar resection (OR = 1.411, 95% CI: 1.006–1.816, *p* = 0.048). An increased number of chest drains was associated with a higher risk of severe pain (OR = 1.221, 95% CI: 1.084–1.358, *p* = 0.045). Higher cough VAS scores were also significantly associated with greater pain severity (OR = 1.241, 95% CI: 1.029–1.496, *p* = 0.024). Furthermore, patients with severe anxiety had a markedly elevated risk of severe BTcP compared to those without anxiety (OR = 20.560, 95% CI: 2.170–194.773, *p* = 0.027).

**TABLE 4:** Multivariate logistic regression analysis of breakthrough pain severity in the resected cohort.

Characteristics	OR	95% CI	*p*
Surgical extent			**0.048**
Sublobectomy	1		
Lobectomy	1.411	1.006–1.816	
Drainage tube count			**0.045**
Continual	1.221	1.084–1.358	
Cough VAS score			**0.024**
Continual	1.241	1.029–1.496	
Anxiety assessment			**0.027**
No	1		
Mild	1.935	0.872–4.296	
Moderate	2.286	0.711–7.351	
Severe	20.560	2.170–194.773	

*Note:* Bold value means p < 0.05. Abbreviations: CI, confidence interval; OR, odds ratio; VAS, visual analog scale.

## 4. Discussion

This study investigated the characteristics and influencing factors of BTcP in 255 lung cancer patients, yielding several important findings: (1) BTcP in lung cancer patients was found to occur frequently, with short duration and moderate to severe intensity. The chest was the most commonly reported site, and cough was identified as a major trigger. These findings highlight the need for clinicians to pay close attention to pain patterns when developing individualized pain management strategies, allowing for timely adjustments to treatment plans. (2) The severity of BTcP was significantly associated with anxiety levels, cough intensity, and treatment modality, indicating that psychological state and symptom control play a critical role in pain management. (3) High levels of anxiety and depression were common among patients with BTcP, and these psychological factors were closely related to both the frequency and severity of pain episodes. This underscores the importance of incorporating psychological interventions into comprehensive pain management approaches.

Our results showed that the severity of BTcP in this cohort was predominantly moderate, with 56.9% (145/255) of patients reporting moderate pain intensity. However, Davies et al. [18] found that among BTcP patients, 34% experienced moderate pain, 62% reported severe pain, and 81% indicated that pain interfered with normal activities. Compared with previous studies [10, 18] that mainly enrolled patients with advanced cancer, our cohort included a relatively high proportion of patients with Stage I disease and those undergoing surgical treatment. This difference may partly explain the lower severity of BTcP observed in our study. Patients with early‐stage disease, particularly those treated surgically, may experience pain that is more closely related to postoperative tissue injury, chest drainage, cough, and functional recovery, rather than the sustained tumor burden, extensive metastasis, or complex systemic symptoms often seen in advanced disease. As a result, the clinical presentation, triggering factors, and severity of BTcP in our cohort may differ from those reported in studies focusing predominantly on advanced cancer populations [10, 18]. Therefore, comparisons between our findings and those of previous studies should be interpreted with caution.

An additional noteworthy finding was that 37.3% (95/255) of patients with BTcP received no analgesic treatment, and among treated patients, weak opioids were more commonly used than strong opioids, despite over half reporting severe peak pain. This apparent mismatch between pain severity and analgesic use may indicate undertreatment of BTcP in clinical practice. Possible explanations include inadequate recognition of transient severe pain episodes, concerns about opioid‐related adverse effects, and insufficient individualized adjustment of rescue analgesia. Moreover, among patients receiving analgesics, 52.5% (84/160) reported an onset time of more than 30 min, whereas 42.4% (108/255) of BTcP episodes lasted less than 5 min, suggesting that conventional oral analgesics may not adequately match the rapid onset and short duration of BTcP episodes. Guidelines and reviews have emphasized that rescue medications for BTcP should ideally provide rapid analgesia with an appropriate duration of action [19, 20]. Therefore, rapid‐onset opioids may be considered for appropriately selected opioid‐tolerant patients with controlled background pain, while patient‐controlled analgesia may be useful in closely monitored inpatient or postoperative settings [20, 21]. For predictable incident pain, such as cough‐ or movement‐related pain, anticipatory analgesia before triggering activities may also help improve pain control [22]. These findings highlighted the need for individualized BTcP management strategies based on pain onset, duration, predictability, opioid tolerance, safety profile, and clinical setting.

We found that anxiety was a significant factor influencing the intensity of BTcP, in both the entire cohort and the postoperative subgroup. Higher anxiety level was associated with more severe BTcP. This is supported by the findings of Annunziata et al. [23], who reported that anxiety lowers pain thresholds and amplifies pain perception, creating a vicious cycle. However, the association between anxiety and BTcP severity should be interpreted with caution, as the relationship is likely bidirectional. More severe pain may contribute to increased anxiety, while anxiety may also heighten pain perception and exacerbate the experience of BTcP. Because of the cross‐sectional design, the temporal and causal relationship between these factors could not be determined. Nevertheless, several plausible mechanisms may help explain the observed association: (1) Neuroendocrine pathway: Anxiety leads to hyperactivation of the hypothalamic–pituitary–adrenal axis, resulting in elevated cortisol levels [24]. Chronic hypercortisolemia impairs the endogenous opioid system, reducing the body’s natural pain regulation. At the same time, anxiety activates the amygdala, heightening sensitivity to pain‐related stimuli and intensifying pain perception [24]. (2) Psychophysiological feedback loop: Anxiety exacerbates pain perception, increasing both the frequency and intensity of BTcP episodes. In turn, repeated pain episodes reinforce anxiety, establishing a self‐perpetuating cycle [25]. (3) Behavioral effects: Anxiety‐related insomnia can significantly lower pain thresholds, making patients more sensitive to nociceptive stimuli. (4) Additionally, anxiety may lead to pain‐avoidant behaviors, which can cause muscle atrophy and joint stiffness, further aggravating pain symptoms [25]. These findings emphasize the importance of integrating psychological support into clinical care for patients with lung cancer experiencing BTcP. Early assessment and intervention for patients at psychological risk may reduce the incidence of breakthrough pain and improve overall quality of life.

Our study also confirmed that cough is not only one of the most common triggers of BTcP but also an independent influencing factor, consistent with previous findings by Mercadante et al. [10]. The potential mechanisms [26, 27] through which cough exacerbates BTcP include the following: (1) Mechanical stimulation: Violent coughing causes strong contractions of the chest wall muscles and diaphragm, directly inducing or aggravating pain—especially when the tumor involves the pleura or chest wall. (2) Inflammatory response: Frequent, forceful coughing may cause microtrauma to peritumoral tissues, triggering localized inflammation and the release of proinflammatory cytokines, which further enhance pain perception. (3) Intrathoracic pressure fluctuations: Coughing‐induced changes in intrathoracic pressure may disrupt blood flow and nerve signaling around the tumor, indirectly intensifying pain. These pressure shifts may also tug on tumor‐associated tissues, directly stimulating nociceptive nerve endings and triggering BTcP. Although cough is a modifiable risk factor for BTcP, it also plays a critical role in postoperative lung cancer recovery by promoting sputum clearance and lung re‐expansion [26]. Therefore, clinicians must strike a balance between effective pain relief and the preservation of productive cough.

In the postoperative subgroup, the number of chest drainage tubes was independently associated with BTcP severity. This association may be explained by several postoperative pain mechanisms. Chest drainage tubes can mechanically irritate the parietal pleura, intercostal nerves, and surrounding chest wall tissues, thereby increasing nociceptive input and predisposing patients to transient pain exacerbations [28, 29]. A greater number of tubes may intensify local mechanical stimulation and aggravate pain during coughing, deep breathing, turning over, or mobilization. In addition, local tissue compression, friction, and inflammatory responses around the insertion sites may increase peripheral sensitization and pain sensitivity [28, 30]. These findings suggested that careful assessment of drainage tube necessity, appropriate tube positioning, and timely pain management may be important for reducing BTcP severity during postoperative recovery.

This study has several limitations. First, although patients were prospectively enrolled, the cross‐sectional design limits causal inference and remains subject to potential bias. Second, the study was conducted at a single center with a limited sample size, which may affect the generalizability and representativeness of the findings. To enhance the applicability and scientific value of the results, future studies should expand the sample size and involve multicenter collaborations across different regions and populations. Third, the assessment of BTcP relied on the English version of the BAT Tool, which, although previously validated, has not been culturally adapted or validated for use in Chinese populations. Further research is needed to translate and validate a Chinese version to ensure its relevance and accuracy in local clinical practice.

## 5. Conclusions

In conclusion, BTcP is common among lung cancer patients and is characterized by high frequency, short duration, and moderate to severe intensity, with the chest as the most common pain site and cough as a major trigger. BTcP severity was associated with treatment modality, cough severity, and anxiety level, underscoring the importance of both symptom control and psychological support. Comprehensive and individualized pain management strategies may help improve pain control and quality of life in this population.

## Author Contributions

(I) Conception and design: Hui‐Ying Qin and Jiu‐Di Zhong.

(II) Administrative support: Hui‐Ying Qin and Jin‐Bo Li.

(III) Methodology: Xiao‐Meng Dou and Jiu‐Di Zhong.

(IV) Investigation: Xiao‐Meng Dou, Bao‐Wen Huang, and Xiao‐Xiao Huang.

(V) Manuscript writing: Xiao‐Meng Dou.

(VI) Funding acquisition: None.

## Funding

No funding was received for this research.

## Disclosure

All authors gave final approval of the manuscript.

## Ethics Statement

This study was approved by the Ethics Committee of the Sun Yat‐Sen University Cancer Center (Ethics Approval No. B2025‐072‐01) and received support and consent from the relevant departments. Written informed consent was obtained only after ensuring full patient understanding.

## Conflicts of Interest

The authors declare no conflicts of interest.

## Supporting Information

Additional supporting information can be found online in the Supporting Information section.

## Supporting information


**Supporting Information** Table S1. Univariate logistic regression analysis of breakthrough pain severity in the entire cohort. Table S2. Collinearity analysis of potential influencing factors incorporated into the multivariate model in the entire cohort. Table S3. Univariate logistic regression analysis of breakthrough pain severity in the resected cohort. Table S4. Collinearity analysis of potential influencing factors incorporated into the multivariate model in the resected cohort. Document S1. The formal Ethical Approval certificate issued by the Ethics Committee of Sun Yat‐Sen University Cancer Center.

## Data Availability

The data that support the findings of this study are available from the corresponding author upon reasonable request.
