# Supplementary material for: Patterns and Associated Factors of Breakthrough Pain in Lung Cancer: Insights From a Cross‐Sectional Study With Prospective Enrollment
Source: Pain Res Manag. 2026 Aug 19;2026:2783248. doi: 10.1155/prm/2783248 (PMC13487466; doi:10.1155/prm/2783248)
Supplement: Supplementary file 1 [file PRM-2026-2783248-s001.zip › Supplement Document S1-Ethics Statement.pdf]

中山大学肿瘤防治中心伦理委员会

文件编号：SL-B2025-072-01

同意函

同意函号：B2025-072-01　　　　同意日期：2025-02-10

项目名称	肺癌患者爆发痛现状调查及影响因素分析
项目负责人	窦晓蒙
项目负责人科室	胸科
审查类别	初始审查
审查方式	快速审查
审查文件目录	见附件
审查意见	本伦理委员会于 2025 年 02 月 10 日严格按照 ICH-GCP 原则及中国相关的法规/指南，由 2 位委员通过快审形式审阅了该项目。审查结果：同意。
年度/定期跟踪审查频率	12 个月
同意函失效日期	2026-02-09

主任委员或副主任委员签名：

中山大学肿瘤防治中心伦理委员会（盖章）

日期：2025 年 02 月 10 日

注意事项：

1. 请遵循《赫尔辛基宣言》（2024 年）、ICH-GCP（2016 年）、《药物临床试验质量管理规范》（2020 年）、《药物临床试验伦理审查工作指导原则》（2010 年）、《涉及人的生命科学和医学研究伦理审查办法》（2023 年）、《涉及人的生物医学研究伦理审查办法》（2016 年）等相关国际准则和国内法律、法规。遵循伦理委员会批准的方案开展临床研究，保护受试者的健康与权利。

2. 研究过程中若变更主要研究者，对临床研究方案、知情同意书、招募材料等的任何修改，请申请人提交修正案审查申请。

3. 发生 SAE/SUSAR，请申请人及时提交。

4. 请按照伦理委员会规定的年度定期跟踪审查频率，申请人在截止日期前 1 个月提交年度定期跟踪审查报告。

5. 申请人暂停或提前终止临床研究，请及时提交暂停/终止研究报告。

6. 完成临床研究，请申请人提交结题报告。

声明：

研究实施前提：

1.所有研究必须有伦理委员会同意函以及 NMPA 正式批件方能实施。若 NMPA 提出意见，则需按照 NMPA 意见进行必要的方案修订并报伦理审批同意后方可实施。

2.研究实施前，请申请人在国家卫生健康委员会“医学研究登记备案信息系统”完成备

中山大学肿瘤防治中心伦理委员会

案。<br>3.如属《人类遗传资源采集、收集、买卖、出口、处境审批行政许可事项》规定范畴的研究，需获得中国人类遗传资源管理部门的批准/备案成功后方可实施。

附件：审查文件目录：

初始审查申请审查目录如下：
1.初始审查申请表
2.研究者发起的立项意见表
3.研究者发起的项目组成人员签名表
4.经济利益声明表
5.本中心主要研究者简历
6.研究方案（版本号：01, 版本日期：2025.01.20）
7.知情同意书（版本号：1.0, 版本日期：2025年01月20日）
8.病例报告表（CRF）（版本号：01, 版本日期：2025.01.20）
9.调查问卷(含一般资料调查表、咳嗽视觉模拟评分和医院焦虑抑郁量表(HADS))（版本号：1.0, 版本日期：2025年01月20日）
10.初始审查申请表签字扫描版

同意函号：B2025-072-01

项目名称：肺癌患者爆发痛现状调查及影响因素分析

中山大学肿瘤防治中心伦理委员会成员名单

伦理委员会职务	姓名	性别	职务/职称	专业	签名
委员	覃惠英	女	护理部主任/主任护师	护理学	（签名）
委员	柳娜	女	实验研究部研究员	肿瘤学	（签名）

日期:2025年02月10日

Ethics Update – May 2026

伦理审查意见

点击附件下载批件意见

文件编号:	SL-B2025-072-03
研究项目名称	肺癌患者爆发痛现状调查及影响因素分析
研究科室	胸科
主要研究者	窦晓蒙
审查类别	研究完成审查
审查方式	快速审查
审查日期	2026-05-20
审查意见	本伦理委员会于2026年05月20日严格按照ICH-GCP原则及中国相关的法规/指南，由1位委员通过快审形式审阅了该项目。审查结果：同意。
日期:	2026-05-20

文件名称	上传文件
