# Supplementary material for: Patterns and Associated Factors of Breakthrough Pain in Lung Cancer: Insights From a Cross‐Sectional Study With Prospective Enrollment
Source: Pain Res Manag. 2026 Aug 19;2026:2783248. doi: 10.1155/prm/2783248 (PMC13487466; doi:10.1155/prm/2783248)
Supplement: Supplementary file 1 [file PRM-2026-2783248-s001.zip › Supplements-Clean version.docx]

Table S1. Univariate logistic regression analysis of breakthrough pain severity in the entire cohort

|  | OR | 95% CI | P |
| --- | --- | --- | --- |
| Age, years |  |  | 0.931 |
| Continual | 0.999 | 0.979-1.020 |  |
| Gender |  |  | 0.812 |
| Male | 1 |  |  |
| Female | 0.812 | 0.491-1.345 |  |
| BMI |  |  | 0.100 |
| Continual | 0.933 | 0.859-1.013 |  |
| Marital status |  |  | 0.191 |
| Married | 1 |  |  |
| Others | 0.082 | -0.242-1.204 |  |
| Ethnicity |  |  | 0.744 |
| Han | 1 |  |  |
| Others | 0.785 | 0.184-3.358 |  |
| Religion |  |  | 0.438 |
| Without | 1 |  |  |
| With | 0.519 | 0.099-2.742 |  |
| Educational level |  |  | 0.975 |
| Primary school or below | 1 |  |  |
| Junior high school | 0.827 | 0.412-1.659 |  |
| Vocational school | 0.771 | 0.290-2.049 |  |
| Senior high school | 0.796 | 0.330-1.917 |  |
| College diploma | 0.694 | 0.256-1.885 |  |
| Bachelor degree | 0.771 | 0.327-1.818 |  |
| Postgraduate degree | 0.360 | 0.035-3.691 |  |
| Residence |  |  | 0.393 |
| Urban | 1 |  |  |
| Rural | 1.248 | 0.751-2.073 |  |
| Occupation |  |  | 0.952 |
| Retired/Unemployed | 1 |  |  |
| Employed | 0.984 | 0.572-1.691 |  |
| Income, Yuan |  |  | 0.484 |
| <=2000 | 1 |  |  |
| 2000-4000 | 0.556 | 0.242-1.276 |  |
| 4000-6000 | 0.793 | 0.333-1.887 |  |
| >=6000 | 0.833 | 0.382-1.818 |  |
| Smoking |  |  | 0.260 |
| Never smoker | 1 |  |  |
| Former smoker | 1.295 | 0.763-2.199 |  |
| Current smoker | 2.275 | 0.770-6.721 |  |
| Drinking history |  |  | 0.727 |
| Without | 1 |  |  |
| With | 0.815 | 0.259-2.565 |  |
| Hypertension |  |  | 0.363 |
| Without | 1 |  |  |
| With | 0.757 | 0.415-1.379 |  |
| Diabetes Mellitus |  |  | 0.300 |
| Without | 1 |  |  |
| With | 1.474 | 0.708-3.067 |  |
| Tumor site |  |  | 0.096 |
| Right | 1 |  |  |
| Left | 0.699 | 0.401-1.219 |  |
| Others | 1.950 | 0.795-4.782 |  |
| TNM stage |  |  | **<0.001** |
| I | 1 |  |  |
| II | 1.138 | 0.277-4.673 |  |
| III | 3.542 | 1.511-8.301 |  |
| IV | 5.210 | 2.794-9.716 |  |
| Undetermined | 1.417 | 0.548-3.661 |  |
| Pathological type |  |  | 0.429 |
| Adenocarcinoma | 1 |  |  |
| Squamous cell carcinoma | 1.296 | 0.648-2.591 |  |
| Others | 0.746 | 0.392-1.420 |  |
| Treatment modality |  |  | **<0.001** |
| Surgery | 1 |  |  |
| Chemotherapy/radiotherapy | 4.549 | 2.647-7.820 |  |
| Baseline pain score |  |  | 0.839 |
| Continue | 1.045 | 0.685-1.595 |  |
| Cough VAS score |  |  | **0.001** |
| Continue | 1.211 | 1.080-1.359 |  |
| Anxiety assessment |  |  | **<0.001** |
| No | 1 |  |  |
| Mild | 2.488 | 1.390-4.452 |  |
| Moderate | 2.885 | 1.293-6.437 |  |
| Severe | 21.636 | 4.735-98.875 |  |
| Depression assessment |  |  | **<0.001** |
| No | 1 |  |  |
| Mild | 1.467 | 0.797-2.700 |  |
| Moderate | 2.829 | 1.390-5.757 |  |
| Severe | 8.271 | 2.850-24.005 |  |

OR, odds ratio; CI, confidence interval; TNM, tumor-node-metastasis; VAS, visual analogue scale

Table S2. Collinearity analysis of potential influencing factors incorporated into multivariate Model in the entire cohort

|  | Tolerance | VIF |
| --- | --- | --- |
| TNM stage | 0.581 | 1.721 |
| Treatment modality | 0.470 | 2.128 |
| Cough VAS score | 0.870 | 1.150 |
| Anxiety assessment | 0.481 | 2.078 |
| Depression assessment | 0.585 | 1.710 |

VIF, variance inflation factor; TNM, tumor-node-metastasis; VAS, visual analogue scale

Table S3. Univariate logistic regression analysis of breakthrough pain severity in the resected cohort

|  | OR | 95% CI | P |
| --- | --- | --- | --- |
| Age, years |  |  | 0.583 |
| Continual | 0.992 | 0.965-1.020 |  |
| Gender |  |  | 0.392 |
| Male | 1 |  |  |
| Female | 0.741 | 0.373-1.472 |  |
| BMI |  |  | 0.984 |
| Continual | 0.999 | 0.883-1.130 |  |
| Marital status |  |  | 0.719 |
| Married | 1 |  |  |
| Others | 0.780 | 0.202-3.020 |  |
| Ethnicity |  |  | 0.437 |
| Han | 1 |  |  |
| Others | 1.841 | 0.396-8.565 |  |
| Religion |  |  | 0.953 |
| Without | 1 |  |  |
| With | 0.951 | 0.178-5.085 |  |
| Educational level |  |  | 1.000 |
| Primary school or below | 1 |  |  |
| Junior high school | 1.236 | 0.418-3.655 |  |
| Vocational school | 1.111 | 0.273-4.520 |  |
| Senior high school | 1.067 | 0.240-4.740 |  |
| College diploma | 1.111 | 0.273-4.520 |  |
| Bachelor degree | 1.037 | 0.288-3.736 |  |
| Postgraduate degree | 0.889 | 0.077-10.300 |  |
| Residence |  |  | 0.374 |
| Urban | 1 |  |  |
| Rural | 1.375 | 0.681-2.779 |  |
| Occupation |  |  | 0.390 |
| Retired/Unemployed | 1 |  |  |
| Employed | 0.732 | 0.360-1.491 |  |
| Income, Yuan |  |  | 0.073 |
| <=2000 | 1 |  |  |
| 2000-4000 | 0.268 | 0.087-0.824 |  |
| 4000-6000 | 0.660 | 0.227-1.923 |  |
| >=6000 | 0.367 | 0.1127-1.060 |  |
| Smoking |  |  | 0.069 |
| Never smoker | 1 |  |  |
| Former smoker | 1.333 | 0.619-2.870 |  |
| Current smoker | 7.143 | 1.311-38.918 |  |
| Drinking history |  |  | 0.975 |
| Without | 1 |  |  |
| With | 1.023 | 0.253-4.137 |  |
| Hypertension |  |  | 0.496 |
| Without | 1 |  |  |
| With | 0.746 | 0.320-1.737 |  |
| Diabetes Mellitus |  |  | 0.460 |
| Without | 1 |  |  |
| With | 1.458 | 0.536-3.971 |  |
| Tumor site |  |  | 0.445 |
| Right | 1 |  |  |
| Left | 0.622 | 0.401-1.219 |  |
| Others | 0.000 | 0.000-0.000 |  |
| Surgical extent |  |  | **<0.001** |
| Sublobectomy | 1 |  |  |
| Lobectomy | 2.358 | 1.600-3.116 |  |
| Surgical duration, hour |  |  | 0.171 |
| 1 | 1 |  |  |
| 2 | 1.037 | 0.432-2.487 |  |
| 3 | 0.346 | 0.116-1.030 |  |
| 4 | 0.376 | 0.107-1.327 |  |
| 5 | 1.339 | 0.261-6.861 |  |
| 6 | 1.786 | 0.226-14.099 |  |
| Drainage tube count |  |  | **<0.001** |
| Continual | 2.455 | 1.316-3.594 |  |
| Duration of drainage tube placement |  |  | 0.870 |
| Continual | 1.026 | 0.755-1.395 |  |
| Time to first ambulation postoperatively |  |  | 0.362 |
| Continual | 0.749 | 0.402-1.395 |  |
| Time to first bowel movement postoperatively |  |  | 0.117 |
| Continual | 0.810 | 0.622-1.054 |  |
| Postoperative complication |  |  | 0.498 |
| Without | 1 |  |  |
| With | 0.578 | 0.118-2.829 |  |
| Lenth of hospital stay, day |  |  | 0.604 |
| Continual | 1.026 | 0.932-1.128 |  |
| Preoperative WBC count, 10⁹/L |  |  | 0.785 |
| Continual | 0.971 | 0.787-1.199 |  |
| Postoperative Day 1 WBC, 10⁹/L |  |  | 0.919 |
| Continual | 1.005 | 0.911-1.108 |  |
| WBC on day before discharge, 10⁹/L |  |  | 0.866 |
| Continual | 1.007 | 0.917-1.094 |  |
| TNM stage |  |  | 0.848 |
| I | 1 |  |  |
| II | 1.594 | 0.360-7.057 |  |
| III | 1.138 | 0.277-4.673 |  |
| IV | 2.656 | 0.161-43.745 |  |
| Undetermined | 1.518 | 0.582-3.960 |  |
| Pathological type |  |  | 0.583 |
| Adenocarcinoma | 1 |  |  |
| Squamous cell carcinoma | 0.651 | 0.198-2.140 |  |
| Others | 0.677 | 0.277-1.651 |  |
| Baseline pain score |  |  | 0.748 |
| Continual | 1.113 | 0.581-2.132 |  |
| Cough VAS score |  |  | **0.018** |
| Continual | 1.241 | 1.038-1.485 |  |
| Anxiety assessment |  |  | **0.021** |
| No | 1 |  |  |
| Mild | 2.049 | 0.941-4.464 |  |
| Moderate | 2.491 | 0.789-7.871 |  |
| Severe | 18.684 | 2.058-169.625 |  |
| Depression assessment |  |  | 0.962 |
| No | 1 |  |  |
| Mild | 1.204 | 0.567-2.554 |  |
| Moderate | 1.231 | 0.387-3.912 |  |
| Severe | 1.441 | 0.236-4.112 |  |

OR, odds ratio; CI, confidence interval; TNM, tumor-node-metastasis; WBC, white blood cell; VAS, visual analogue scale

Table S4. Collinearity analysis of potential influencing factors incorporated into multivariate Model in the resected cohort

|  | Tolerance | VIF |
| --- | --- | --- |
| Surgical extent | 0.914 | 1.094 |
| Drainage tube count | 0.912 | 1.097 |
| Cough VAS score | 0.994 | 1.006 |
| Anxiety assessment | 0.992 | 1.008 |

VIF, variance inflation factor; VAS, visual analogue scale
